# Growth Pattern and Condition in the Mudskipper *Scartelaos histophorus* in the Mekong Delta

**DOI:** 10.1002/ece3.73028

**Published:** 2026-02-04

**Authors:** Gieo Hoang Phan, Quang Minh Dinh, Ton Huu Duc Nguyen

**Affiliations:** ^1^ Faculty of Agriculture and Rural Development Kien Giang University An Giang Vietnam; ^2^ Faculty of Biology Education, School of Education Can Tho University Can Tho Vietnam

**Keywords:** allometry, condition factor, length–weight relationship, *Scartelaos histophorus*

## Abstract

Studies of the length–weight relationship (LWR) and condition factor (CF) in fish are abundant but often descriptive; yet, most studies overlook how intrinsic and extrinsic drivers structure these metrics. Here, this study tested whether the growth exponent (*b*) varies across sex, season, and ecological region, and whether CF is elevated in females and before spawning. Over the course of a complete annual cycle, a total of 1436 individuals were collected from four mudflat sites, measured for total length (TL) and weight (W), and analyzed using log10‐linear regressions of LWR and CF, along with appropriate parametric or non‐parametric tests under false discovery rate control. The TL strongly predicted W (*r* ^2^ = 0.87) with *b* = 2.46 ± 0.02 SE, (< 3; *p* < 0.001), indicating negative allometry. Females showed a larger size and higher *b* value (2.52) than males (2.41). The dry season yielded a higher *b* value than the wet season, and southern sites showed a non‐significant trend towards a higher *b* value than northern sites. Mean CF was 1.01 ± 0.01 SE, elevated in females (1.09 vs. 0.98) and in the dry season (1.04 vs. 0.99), while monthly fluctuations (0.89–1.08) tracked feeding and reproduction but showed no regional differences. Overall, *S. histophorus* exhibits consistently negative allometric growth, with systematic variation in *b* and CF across sex and season. These findings provide hypothesis‐driven baselines for monitoring semi‐terrestrial gobies and highlight the importance of considering life‐history and hydrological context when applying LWR/CF in ecological and evolutionary research and management.

## Graphical abstract

Analysis of 1436 mudskippers ( *Scartelaos histophorus* ) from the Mekong Delta revealed consistently negative allometric growth. Growth exponents and condition factors were higher in females and during the dry season, emphasizing the roles of life‐history and hydrological drivers in estuarine adaptation.

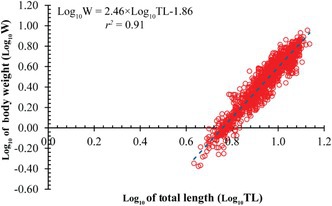

## 1 Introduction

Mudskippers (family Oxudercidae, order Gobiiformes) represent a striking example of amphibious adaptation, thriving in intertidal mudflats and estuaries where they must balance aquatic respiration with aerial exposure. With over 40 described species distributed across tropical and subtropical coasts (Hoese et al. 2006; Froese and Pauly 2025), they display unique traits, cutaneous respiration, terrestrial locomotion, and burrow dwelling, which make them model organisms for studying evolutionary transitions and ecological resilience. Among them, *Scartelaos histophorus* occurs widely in Vietnam, inhabiting mudflats and mangroves where it contributes to ecosystem processes such as sediment aeration and benthic algal regulation, and also holds cultural and subsistence value in the Mekong Delta (Clayton 1993; Graham 1997). Despite its ecological importance and accessibility, little is known about its growth dynamics.

Length–weight relationship (LWR) and condition factor (CF) are fundamental indices in fish biology, linking morphology, energy allocation, and environmental context. The LWR exponent (*b*) reflects growth strategy, indicating whether individuals allocate disproportionately to length or weight, while CF gauges robustness and energy reserves (Le Cren 1951; Ricker 1973; Froese 2006). These indices are widely used in stock assessment, aquaculture, and conservation, but most studies remain descriptive, reporting pooled estimates without explicitly testing how intrinsic and extrinsic factors structure variation (Blackwell et al. 2000; Muchlisin et al. 2010). Yet, theory and prior work suggest that *b* and CF should respond predictably to intrinsic factors such as sex and reproductive stage, as well as extrinsic drivers, including season and habitat (Khallaf et al. 2003; Anene 2005; Faruque and Das 2024). Although LWR and CF are widely applied, most studies report pooled estimates without testing how intrinsic and extrinsic factors shape variation. This descriptive focus overlooks predictable drivers linked to sex, reproductive investment, hydrological cycles, and regional habitat heterogeneity, which recent research indicates can strongly influence allometric growth and condition. For semi‐terrestrial mudskippers, no study has evaluated these mechanisms using a hypothesis‐driven framework across temporal and ecological gradients in the Mekong Delta.

The Mekong Delta is a dynamic estuarine system governed by distinct wet and dry seasons, driven by the southwest and northeast monsoons, and it experiences large tidal fluctuations (3.0–3.5 m) and saltwater intrusion during the dry season (MRC 2005). Coastal mudflats and mangrove forests are vital habitats for amphibious gobies, with *S. histophorus* inhabiting soft mudflats and being entirely dependent on tidal rhythms; this species often occurs in low‐tide zones with clay‐rich mud (Ly et al. 2025). However, canal excavation and land conversion for agriculture and shrimp farming have removed about 50,000 ha of mangroves since 1983–1995, accompanied by heavy pesticide and fertilizer use (Landos 2024); the intensive tra catfish industry also releases 31,602–50,364 t of nitrogen and 9893–15,766 t of phosphorus into the river system each year (De Silva et al. 2010), increasing nutrient loads. These natural dynamics and anthropogenic pressures make the Mekong Delta an important natural laboratory for studying how environmental gradients and fish physiology influence length‐weight relationships and condition factor, as these indicators fluctuate with fishing intensity, ecological stress, and food availability.

Therefore, this study aims to test whether (i) the LWR exponent (*b*) of *S. histophorus* varies across sex, season, and ecological region; (ii) the condition factor (CF) is elevated in females and before spawning; and (iii) regional differences persist after accounting for the nested structure of individuals within months within sites. These predictions align with ecological theory. In fishes, females typically allocate substantial energetic reserves to oocyte development and gonad maturation, resulting in elevated CF before spawning as somatic resources are reallocated into reproductive tissues rather than external growth (Farley et al. 2015). This pattern has been widely documented in gobiids and other estuarine taxa, where reproductive investment drives sex‐specific differences in condition (Utne‐Palm et al. 2015). Similarly, regional differences may emerge if habitat quality varies across the Hau River boundary. Northern and southern mudflat systems differ in their hydrodynamics, sediment profiles, and freshwater intrusion, which in turn influence food availability, metabolic stress, and burrow stability. Consequently, populations inhabiting distinct ecological regions may exhibit divergent growth scaling and energetic strategies (Faruque and Das 2024). This study takes a hypothesis‐driven approach to growth biology in *S. histophorus* . Using monthly collections from four sites across the Mekong Delta over a year, this study tests these predictions and establishes baselines for understanding how life‐history stage and hydrology shape morphology–weight scaling and condition in an amphibious fish. By embedding LWR and CF within an inferential framework, the study moves beyond description and provides insights relevant to ecological theory and the sustainable management of estuarine resources.

## 2 Material and Method

### 2.1 Study Area and Sampling Design

**FIGURE 1:**
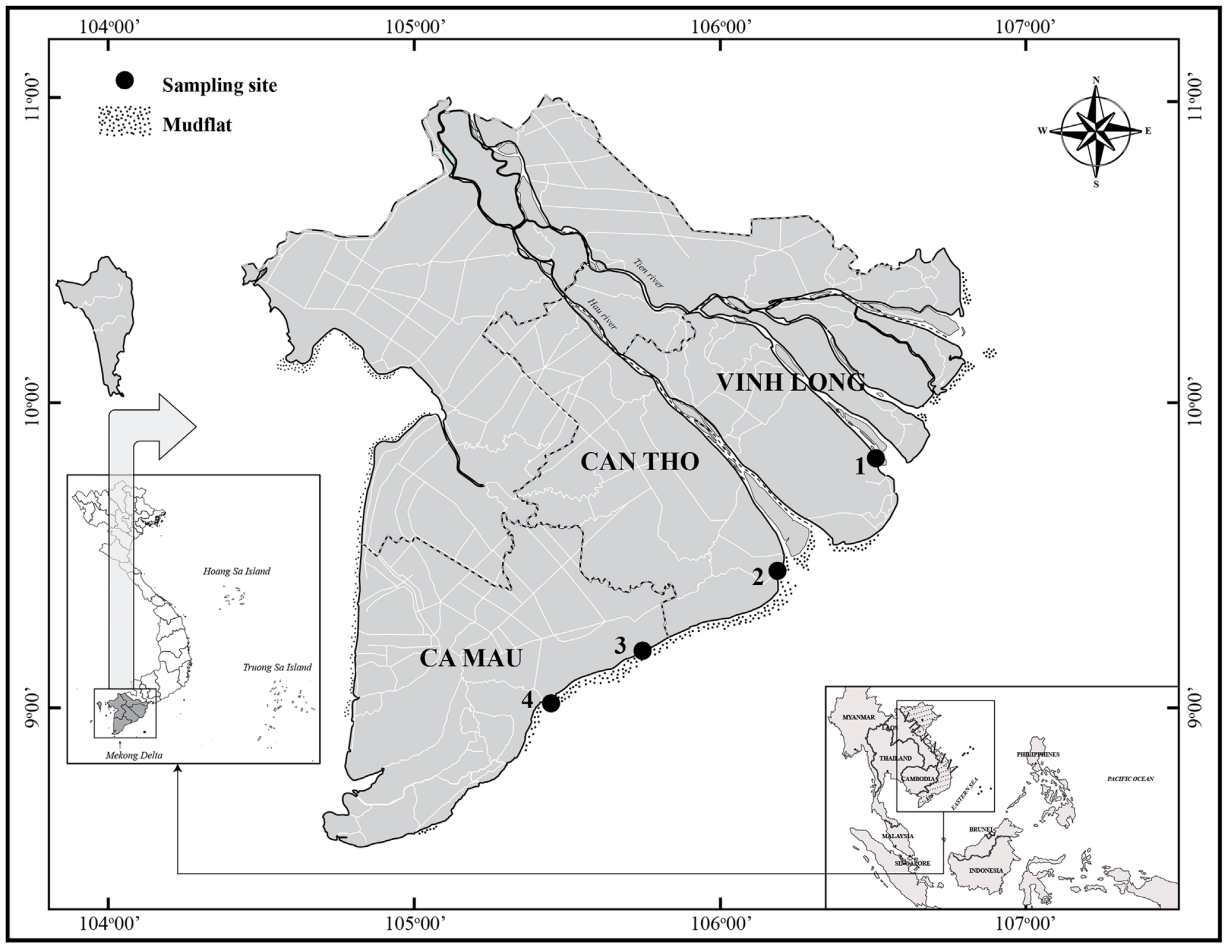
Sampling sites of *Scartelaos histophorus* in the Mekong Delta: (1) My Long–Vinh Long, (2) Tran De–Can Tho, (3) Hiep Thanh–Ca Mau, and (4) Ganh Hao–Ca Mau. Map modified from Dinh (2018).

To test for sex‐, season‐, and region‐specific growth and condition drivers, this study established a year‐round sampling program at four mudflat sites in the Mekong Delta, Viet Nam. Two sites were located north of the Hau River (BSH: My Long, Vinh Long—9°33′19.0 ″N 106°22′06.0″E; Tran De, Can Tho—9°26′19.7″N 106°10′55.2″ E) and two south of the Hau River (NSH: Hiep Thanh—9°12′08.0″N 105°44′36.0″ E and Ganh Hao, Ca Mau—9°01′47.0″N 105°25′55.0″ E; Figure 1). Sites were chosen to represent contrasting ecological regions while minimizing the confounding effects of distance.

Sampling was conducted on the lowest tidal day of each month from August 2024 to July 2025 to ensure maximum access to burrows. At each site, ~100 m^2^ of mudflat was surveyed, and individuals were randomly hand‐collected by excavating shallow burrows at ebb tide. Approximately 30 individuals were collected at each site each month (Table 1). Specimens were anesthetized with Finquel MS‐222 and fixed in 10% formalin (1:9 dilution). All animal procedures were approved by the Animal Ethics Committee of Can Tho University (CTU‐AEC24007) and complied with institutional and national regulations.

**TABLE 1:** Monthly variation in growth parameters and condition factor of *S. histophorus* from August 2024 to July 2025.

Month	*N*	TL (mean ± SE)	*W* (mean ± SE)	*b* (mean ± SE)	*a* (mean ± SE)	*r* ^2^	*t*_*s*_	*p*	Type	CF (mean ± SE)
Aug‐24	103	8.7 ± 0.2	3.17 ± 0.15	2.23 ± 0.07	0.024 ± 0.004	0.88	−11.03	0.00	N	1.08 ± 0.02
Sep‐24	141	10.4 ± 0.1	4.53 ± 0.10	1.98 ± 0.10	0.044 ± 0.010	0.69	−10.34	0.00	N	1.05 ± 0.01
Oct‐24	90	10.3 ± 0.2	4.13 ± 0.15	2.51 ± 0.13	0.011 ± 0.003	0.71	−3.88	0.00	N	0.96 ± 0.02
Nov‐24	101	9.8 ± 0.2	4.05 ± 0.13	2.13 ± 0.06	0.030 ± 0.004	0.90	−15.00	0.00	N	1.05 ± 0.01
Dec‐24	159	7.6 ± 0.2	2.14 ± 0.13	2.35 ± 0.05	0.016 ± 0.001	0.90	−14.15	0.00	N	0.94 ± 0.01
Jan‐25	127	8.1 ± 0.2	2.76 ± 0.15	2.60 ± 0.07	0.011 ± 0.002	0.94	−6.12	0.00	N	1.08 ± 0.02
Feb‐25	92	8.8 ± 0.2	3.24 ± 0.15	2.53 ± 0.07	0.013 ± 0.002	0.89	−6.41	0.00	N	1.08 ± 0.01
Mar‐25	103	9.0 ± 0.2	3.33 ± 0.15	2.39 ± 0.07	0.016 ± 0.002	0.93	−9.40	0.00	N	1.04 ± 0.01
Apr‐25	131	9.0 ± 0.1	3.08 ± 0.13	2.62 ± 0.10	0.009 ± 0.002	0.81	−3.88	0.00	N	0.97 ± 0.02
May‐25	159	8.5 ± 0.1	2.98 ± 0.13	2.58 ± 0.05	0.011 ± 0.001	0.94	−9.31	0.00	N	1.04 ± 0.01
Jun‐25	132	9.1 ± 0.1	2.95 ± 0.11	2.78 ± 0.10	0.006 ± 0.001	0.77	−2.09	0.04	N	0.89 ± 0.01
Jul‐25	98	8.5 ± 0.2	2.99 ± 0.16	2.74 ± 0.07	0.008 ± 0.001	0.87	−3.56	0.00	N	1.04 ± 0.02
Total	1436	8.9 ± 0.1	3.23 ± 0.04	2.46 ± 0.02	0.014 ± 0.001	0.87	−27.1	0.00	N	1.01 ± 0.01

### 2.2 Morphometric Measurement

In the Zoology laboratory, fish were rinsed in freshwater to remove formalin before measurement. Total length (TL; nearest 0.1 cm) was recorded from snout to caudal tip (Daud et al. 2005), and body weight (W; nearest 0.01 g) was measured using an analytical balance (AND EK‐610). *S. histophorus* exhibits evident external sexual dimorphism. Males possess a triangular and elongated genital papilla, while females have a shorter, oval‐shaped papilla. These traits were well established for Oxudercidae and have been applied in studies of mudskippers from the Mekong Delta (Mahadevan et al. 2019; Dinh, Nguyen, Truong, and Nguyen‐Ngoc 2022). All individuals were sexed based on the morphology of their genital papillae under stereomicroscope observation.

### 2.3 Length–Weight Relationship

The LWR was estimated following Ricker (1973): W = *a* × TL^*b*^, where *a* is the intercept and *b* the scaling exponent. Growth form was classified as isometric (*b* = 3), positive allometry (*b* > 3), or negative allometry (*b* < 3) (Martin 1949). To linearize the relationship, TL and W were log10‐transformed: log_10_(W) = *b* × log_10_(TL) + log_10_(*a*) (Zar 1984).

### 2.4 Condition Factor

Condition factor was quantified using the equation of Le Cren (1951): CF = W/(*a* × TL^*b*^), where *a* and *b* are regression parameters estimated from the LWR for the corresponding group. This calculates the ratio of observed weight to the weight predicted by the LWR, where a value of 1 indicates the individual is of average condition for its length. This formulation standardizes body weight relative to expected values at a given length, thereby providing an index of robustness, energy reserves, and physiological status comparable across sexes, seasons, and regions.

### 2.5 Statistical Analyses

This study tested the hypotheses that growth scaling (*b*) and CF vary by sex, season, and region, and that monthly fluctuations track reproductive and feeding cycles. The TL, W, and CF distributions were assessed for normality (using the Shapiro–Wilk test) and homogeneity of variances. Parametric tests (*t*‐tests, one‐way ANOVA with Tukey HSD) were applied when assumptions were met; otherwise, Mann–Whitney and Kruskal–Wallis tests were used (Kim 2015). Monthly variation was evaluated with ANOVA or non‐parametric equivalents. The exponent *b* was compared against isometry (*b* = 3) using one‐sample *t*‐tests (Mahmood et al. 2012). All analyses were conducted in Jamovi v2.6.44 with significance set at *p* < 0.05. To limit Type I error across multiple contrasts, the Benjamini–Hochberg false discovery rate procedure was applied (Benjamini and Hochberg 1995; McDonald 2014).

## 3 Results

### 3.1 Sample Structure and Morphometrics

A total of 1436 individuals of *S. histophorus* (391 females, 1045 males) were collected across two ecological regions of the Mekong Delta. The sex ratio skew likely reflects natural behavioral differences, as males are more visible and active around burrows during low tide, which increases their capture probability. The TL and W departed significantly from normality (Shapiro–Wilk: TL *W* = 0.98, *p* < 0.001; W *W* = 0.97, *p* < 0.001). The overall mean TL was 8.9 ± 0.1 cm, and mean W was 3.23 ± 0.04 g. Sexual dimorphism was evident: females attained greater TL (9.3 ± 0.1 cm vs. 8.8 ± 0.1 cm; Mann–Whitney *Z* = −4.28, *p* < 0.001) and W (3.70 ± 0.07 g vs. 3.05 ± 0.05 g; *Z* = −7.28, *p* < 0.001). Seasonal analysis showed wet‐season fish were larger (9.1 ± 0.1 cm) and heavier (3.36 ± 0.06 g) than dry‐season fish (8.6 ± 0.1 cm; 3.05 ± 0.06 g; *Z*_TL_ = −5.62, *p* < 0.001; *Z*_*W*_ = −4.06, *p* < 0.001). Regional contrasts indicated that NSH individuals were longer (9.7 ± 0.1 cm) than BSH individuals (8.6 ± 0.1 cm; *Z* = −10.06, *p* < 0.001), whereas BSH individuals were heavier at comparable lengths (3.86 ± 0.08 g vs. 2.96 ± 0.05 g; *Z* = −9.47, *p* < 0.001). Monthly comparisons confirmed significant temporal variation (Kruskal–Wallis: *χ* ^2^_TL_ = 265.75, *p* < 0.001; *χ* ^2^_W_ = 257.68, *p* < 0.001), with maximum size in September 2024 (10.4 ± 0.1 cm; 4.53 ± 0.10 g) and minimum in December 2024 (7.6 ± 0.2 cm; 2.14 ± 0.13 g) (Table 1).

### 3.2 Length–Weight Relationships

**FIGURE 2:**
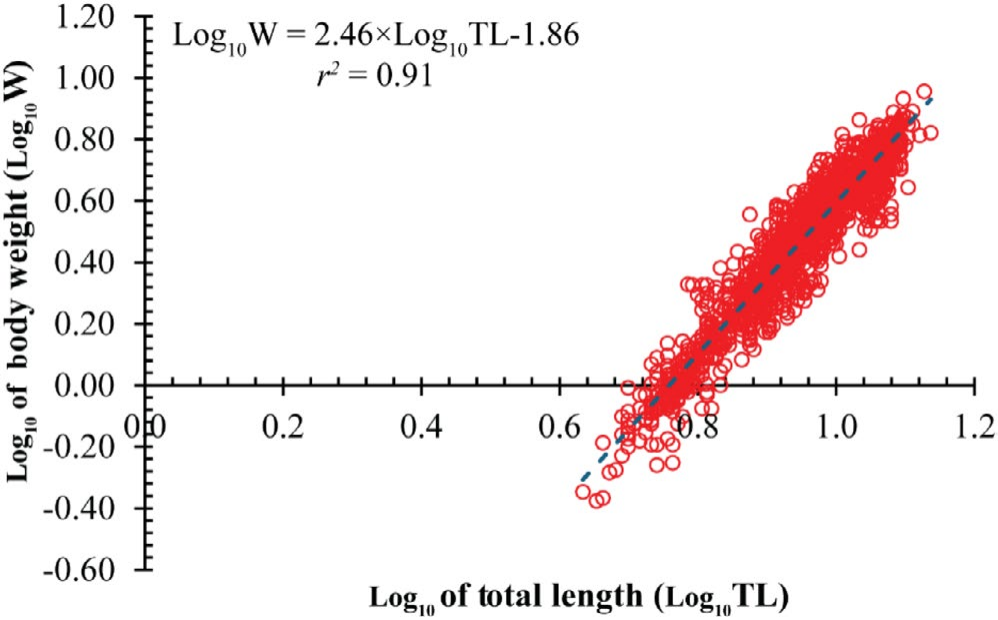
Overall length–weight relationship of *S. histophorus* across the Mekong Delta.

**FIGURE 3:**
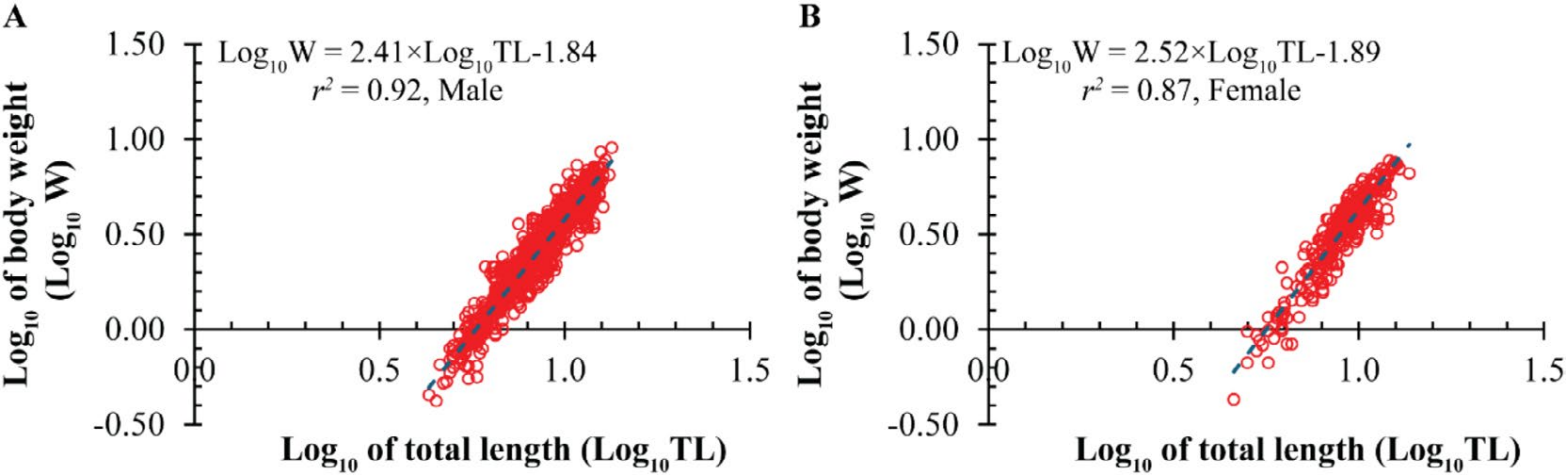
Sex‐specific length–weight relationships of *S. histophorus* : (A) males and (B) females.

**FIGURE 4:**
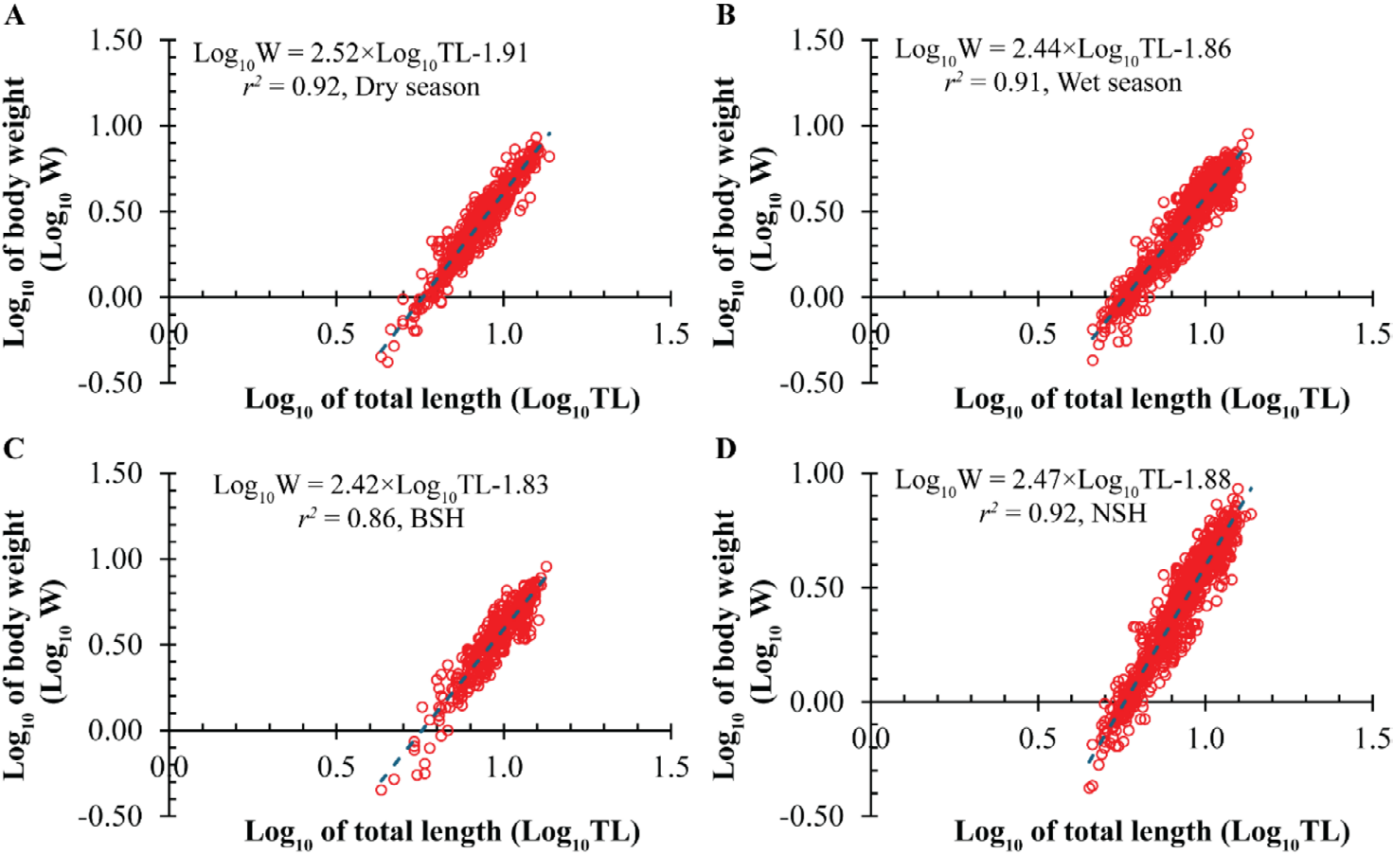
Seasonal and regional length–weight relationships of *S. histophorus* : (A) dry season, (B) wet season, (C) BSH region, and (D) NSH region.

Across the population, TL strongly predicted W (*r* ^2^ = 0.87), with an estimated *b* = 2.46 ± 0.02 SE, significantly < 3 (*t* = −27.10, *p* < 0.001), confirming negative allometry (Figure 2). Sex‐specific slopes differed: males log_10_(W) = 2.41 × log10(TL)−1.84 (*r* ^2^ = 0.92; *t* = −26.64, *p* < 0.001; Figure 3A), females log_10_(W) = 2.52 × log_10_(TL)−1.89 (*r* ^2^ = 0.87; *t* = −10.04, *p* < 0.001; Figure 3B), with females showing slightly higher *b*. Seasonal analyses showed dry‐season fish with *b* = 2.52 (log_10_(W) = 2.52 × log_10_(TL)−1.91; *r* ^2^ = 0.92; *t* = −15.42, *p* < 0.001; Figure 4A) compared to wet‐season fish with *b* = 2.44 (log_10_(W) = 2.44 × log_10_(TL)−1.86; *r* ^2^ = 0.91; *t* = −20.74, *p* < 0.001 Figure 4B). Regionally, BSH fish exhibited *b* = 2.42 (log_10_(W) = 2.42 × log_10_(TL)−1.83; *r* ^2^ = 0.86; *t* = −11.84, *p* < 0.001; Figure 4C), while NSH fish had *b* = 2.47 (log_10_(W) = 2.47 × log_10_(TL)−1.88; *r* ^2^ = 0.92; *t* = −23.04, *p* < 0.001; Figure 4D). Monthly analyses consistently showed negative allometry, with *b* values ranging from 1.98 ± 0.10 in September to 2.78 ± 0.10 in June, and *r* ^2^ values ranging from 0.69 to 0.94 (Table 1).

### 3.3 Condition Factor

The overall CF was 1.01 ± 0.01 SE (Shapiro–Wilk *W* = 0.98, *p* < 0.001). Females exhibited significantly higher CF (1.09 ± 0.01) than males (0.98 ± 0.01; *Z* = −11.13, *p* < 0.001). CF was also higher in the dry season (1.04 ± 0.01) compared to the wet season (0.99 ± 0.01; *Z* = −4.69, *p* < 0.001). No significant differences were observed between regions (NSH 1.01 ± 0.01; BSH 1.01 ± 0.01; *p* = 0.78). Monthly CF fluctuated between 0.89 ± 0.01 (June) and 1.08 ± 0.02 (August), with peaks coinciding with reproductive activity, but values remained consistently close to 1, indicating an overall stable population condition.

## 4 Discussion

This study provides the first hypothesis‐driven evidence that growth and condition in *Scartelaos histophorus* are structured by sex and season in the Mekong Delta. Across 1436 individuals, the scaling exponent *b* was consistently < 3, confirming a negative allometric pattern in which length increases more rapidly than weight. This pattern reflects an energetic trade‐off between structural elongation and tissue accumulation. Increasing body length is comparatively “cheap,” as it primarily involves skeletal extension that facilitates locomotion, burrow excavation, and air‐phase maintenance. In contrast, increasing mass is “expensive,” requiring sustained energy storage in muscle or visceral tissues. Negative allometry, therefore, suggests that selection favors a long, thin morphology over heavier bodies in intertidal environments, where mobility, burrow efficiency, and gas exchange are key determinants of fitness (Mehta et al. 2010). Such disproportionate length growth is consistent with the elongated body form and semi‐terrestrial lifestyle of mudskippers, where efficient locomotion on mudflats, burrow construction, and aerial respiration are more critical for survival than rapid weight accumulation (Murdy 1989; Lee et al. 2005). Females showed both larger body size and higher *b* than males, supporting the hypothesis that sexual dimorphism reflects reproductive investment and greater energy storage during gonadal maturation, as previously observed in other oxudercids (Townsend and Tibbetts 2005). In addition, the apparent male bias may reflect the species' ability to detect by sex. Male mudskippers frequently emerge to defend territories and maintain burrows during low tide, increasing their visibility and the probability of being caught. In contrast, females tend to stay deeper in burrows outside the breeding period, reducing their chance of being caught by hand collection. Therefore, the skewed sex ratios in our dataset should be interpreted with caution and are not necessarily evidence of demographic imbalance or higher mortality among males (Murdy 1989).

The Mekong Delta experiences a pronounced wet–dry cycle, characterized by rainfall brought by the southwest monsoon, which produces a wet season from May to October, and the northeast monsoon, which creates a drier period from November to April (MRC 2005). Meteorological data from nine weather stations indicate that the wet season has a higher average daily temperature, significantly more rainfall and humidity, but lower evaporation and sunshine hours compared to the dry season (Le et al. 2024). These conditions increase freshwater discharge and nutrient availability, dilute salinity, and enhance productivity. Accordingly, individuals collected in the wet season were longer and heavier, suggesting that fish invest energy in structural growth when resources are abundant. By contrast, the dry season is marked by low river flow, high evaporation and intense salt intrusion; each dry season pushes saltwater further inland (Bui 2017; Le and Nguyen 2025), and during severe droughts in 2016 and 2020 salinity levels of about 4 g L^−1^ penetrated 60–78 km inland while temperatures reached 38°C–40°C (Park et al. 2022). This supports the view that length and condition respond differently to environmental drivers: structural growth dominates in resource‐rich wet periods, while energetic reserves accumulate under dry‐season stability (Le Cren 1951; Khallaf et al. 2003). Additionally, in the Mekong Delta, seasonal hydrology significantly influences energy allocation in *S. histophorus* . During the wet season, high freshwater discharge and nutrient loading enhance the biomass of microphytobenthos on exposed mudflats (Jacobs et al. 2021; MRC 2022), creating favorable feeding conditions that support structural growth, as reflected in the larger individuals observed during this period. In contrast, the dry season is characterized by more stable tidal regimes and persistent salinity intrusion, encouraging a shift from somatic elongation to energy storage, consistent with the higher *b* and CF values observed. Accumulation of fat and energy reserves before spawning is a common mechanism in many fish species; for example, studies of black cod in the northeastern United States have shown that spawning is an energy‐consuming process, and fish mobilize liver and muscle reserves for egg development (Slesinger et al. 2022). This pattern aligns with other semi‐terrestrial oxudercids, where pre‐reproductive capital accumulation is a common strategy in fluctuating estuarine systems (Froese 2006; Dinh, Nguyen, Truong, and Nguyen‐Ngoc 2022).

Comparative evidence highlights the plasticity of growth strategies within Oxudercidae, reflecting adaptations to diverse ecological niches, including varying salinity, habitat heterogeneity, and resource availability in intertidal zones (Froese 2006). The negative allometry in *S. histophorus* (*b* = 2.46 ± 0.02 < 3) parallels reports from the Dhamara estuary, India (Mahadevan et al. 2017), and other gobiids in the Mekong, such as *Periophthalmus gracilis* (*b* = 2.69 ± 0.06 < 3, with variations from negative allometry in dry season/immature stages to isometry in wet season/mature stages) (Dinh, Nguyen, Truong, and Nguyen‐Ngoc 2022) and *Acentrogobius viridipunctatus* (*b* = 2.64 ± 0.03 < 3, consistent across sexes/sizes/seasons but varying monthly from negative to isometry) (Dinh, Nguyen, Nguyen‐Ngoc, and Nguyen 2022). The negative variation in *S. histophorus* is also consistent with the mud abalone *Boleophthalmus pectinirostris* from the Maro estuary: the *b* value of males is 2.8562–2.8885, indicating slow weight gain for males, while females have *b* = 3.0907–3.1211, indicating positive growth with a fat body (Sunarni et al. 2019). In contrast, congeners like *P. chrysospilos* (*b* = 3.22 ± 0.02 > 3, with temporal variations) (Dinh, Nguyen, Truong, Tran, and Nguyen 2022) and *P. variabilis* (*b* = 3.094 ± 0.045 > 3, shifting to isometry in wet season/northern sites and positive in dry season/southern sites) (Dinh, Nguyen, Nguyen, Tran, and Truong 2022) exhibit positive allometry, while *Periophthalmodon septemradiatus* grows isometrically (*b* = 3.06 ± 0.01) (Dinh, Nguyen, Truong, Doan, and Nguyen 2022). These discrepancies indicate that growth scaling is not conserved at the family level, but rather modulated by ecological niches, habitat heterogeneity, and reproductive strategies (Froese 2006).

Condition factor analyses provided further support for the above hypotheses. Mean CF near unity indicated overall stable conditions, but significant sex and seasonal differences emerged. Females maintained higher CF than males (1.09 vs. 0.98), reflecting greater somatic reserves for reproduction, a pattern consistent with other fishes from the Mekong (Lam and Dinh 2021; Phan et al. 2021). During the dry season, low river water levels and steady tides make prey more concentrated; studies of snook in Florida showed that body condition increased when prey biomass was high, water levels were low, and during the transition between the wet and dry seasons (Massie et al. 2025), suggesting that low water levels allowed fish to hunt more effectively. Seasonal differences were also pronounced: CF was higher in the dry season (1.04) than in the wet (0.99), suggesting that stable hydrological conditions favor efficient feeding and body weight accumulation. In contrast, the rainy season in the Mekong Delta is characterized by heavy rainfall and strong flows; abundant food promotes length growth but may reduce CF because energy is prioritized for structural development rather than storage (MRC 2022). Interestingly, this trend contrasted with that of *P. gracilis* and *P. chrysospilos* , where CF peaks during the wet season (Abdullah and Zain 2019; Dinh, Nguyen, Truong, and Nguyen‐Ngoc 2022; Dinh, Nguyen, Truong, Tran, and Nguyen 2022). Such contrasts underscore that the condition reflects species‐specific ecological strategies rather than a universal seasonal response.

Despite differences in length and weight, regional comparisons did not reveal significant differences in CF between NSH and BSH (~1.01), suggesting that nutritional quality and environmental support are broadly similar across regions. The absence of regional CF divergence contrasts with findings for *P. septemradiatus* (Dinh, Nguyen, Truong, et al. 2022) and *P. gracilis* (Dinh, Nguyen, Truong, and Nguyen‐Ngoc 2022), where regional habitat heterogeneity drove CF variation. Finally, monthly CF fluctuations (0.89–1.08) mirrored reproductive cycles, peaking before spawning and declining afterward, consistent with mudskippers in other tropical estuaries (Sunarni et al. 2019).

Together, these results confirm that growth and condition in *S. histophorus* are not static descriptors but dynamic traits shaped by sex and season. The LWR analysis showed that this species had negative growth (*b* < 3), but *b* values varied by season and sex: females had higher b than males, and *b* was larger in the dry season than in the wet season; CF was also higher in females and in the dry season, reflecting energy reserves for reproduction. These differences illustrate an adaptive strategy for the intertidal environment, where fish must balance length growth and energy accumulation. Comparative evidence from other semi‐terrestrial gobies supports this finding: *P. gracilis* in the Mekong Delta had an average *b* of 2.69 ± 0.06 (negative growth) but *b* values increased in adults and during the wet season; CF of this species also varied by sex (1.09 in females vs. 0.96 in males) and season, being higher in the wet season (1.05 ± 0.02) than in the dry season (0.99 ± 0.01) (Dinh, Nguyen, Truong, and Nguyen‐Ngoc 2022). Meanwhile, *P. variabilis* exhibited isometric to positive growth (*b* ranging from 2.85 ± 0.10 to 3.37 ± 0.11) and *b* increased significantly in the dry season (3.138 ± 0.065) than in the rainy season (3.058 ± 0.061), but CF of this species (~1.05 ± 0.02) was not sex‐ or season‐dependent but was dominated by location and month (Dinh, Nguyen, Nguyen, Tran, and Truong 2022). These comparative evidences, together with Froese's (2006) review, indicate that biological and spatiotemporal variables influence growth patterns and CF. Le Cren (1951) and Jisr et al. (2018) also suggested that CF fluctuates due to exploitation intensity, environmental stress, and food availability, clarifying that these indices are dynamic measures reflecting physiological status and environmental conditions. By integrating intrinsic and extrinsic drivers, this study extends the utility of LWR and CF from descriptive indices to ecological indicators, providing baseline data for monitoring estuarine fishes and testing broader hypotheses on life‐history adaptation and energy allocation. From a management perspective, the sex‐ and season‐specific variation in *b* and CF indicates that LWR‐derived indicators cannot be treated as fixed species parameters; instead, they should be monitored across hydrological cycles. Because *S. histophorus* relies on stable intertidal mudflats for burrow construction and feeding, degradation of these habitats (e.g., shoreline hardening, mangrove clearance, aquaculture effluent) can disrupt energy allocation and reduce condition. Seasonal shifts in CF, particularly dry‐season reserve accumulation, may therefore serve as early warning signals of environmental stress, complementing abiotic monitoring such as salinity or sediment exposure time. Protecting natural mudflat–mangrove mosaics and maintaining hydrological connectivity are essential to sustaining physiological performance and reproductive success in semi‐terrestrial gobies and other estuarine fishes.

## 5 Conclusion

By integrating sex, season, and regional contrasts, this study provides the first hypothesis‐driven evidence of how growth and condition in *Scartelaos histophorus* are structured in the Mekong Delta. Instead of being static descriptors, the LWR exponent and condition factor emerge as dynamic traits shaped by key drivers: (i) intrinsic life‐history processes such as sexual dimorphism and reproductive investment, which elevate energetic reserves in females; and (ii) seasonal hydrological regimes, where wet‐season productivity promotes structural growth, while dry‐season stability favors weight accumulation and somatic condition. The lack of strong regional divergence suggests that local habitat variability is secondary to physiological and seasonal controls, indicating that energy allocation strategies are conserved primarily across mudflat systems of the Delta. These findings reframe LWR and CF as ecological indicators that integrate physiology, behavioral ecology, and tidal exposure, rather than simple metrics of population status, moving. More broadly, they emphasize the importance of considering both intrinsic and extrinsic drivers when interpreting LWR and CF, and they establish a comparative framework for evaluating adaptive strategies of amphibious gobies across tropical and subtropical estuaries. Future research should combine LWR and CF based assessments with physiological and biochemical indicators to clarify the mechanisms of energy allocation in *S. histophorus* . Long‐term monitoring across multiple hydrological cycles and disturbance gradients would further support the sustainable management of this species and the resilience of intertidal mudflat ecosystems.

## Author Contributions

**Gieo Hoang Phan:** investigation (equal), methodology (equal), writing – original draft (equal), writing – review and editing (equal). **Quang Minh Dinh:** conceptualization (equal), funding acquisition (equal), investigation (equal), methodology (equal), writing – original draft (equal), writing – review and editing (equal). **Ton Huu Duc Nguyen:** investigation (equal), methodology (equal), writing – original draft (equal), writing – review and editing (equal).

## Funding

This work is funded by the Vietnam National Foundation for Science and Technology Development (NAFOSTED) under grant number 106.05‐2023.02.

## Ethics Statement

The study was approved by the Animal Ethics Committee of Can Tho University (CTU‐AEC24007) and was carried out in accordance with the institutional guidelines for the care and use of laboratory animals and the national regulations on animal welfare in Vietnam.

## Conflicts of Interest

The authors declare no conflicts of interest.

## Supporting information


Data S1

## Data Availability

Data was uploaded to the journal system as Supporting Information for review and publication.
